# Enhanced osteogenic and angiogenic capabilities of adipose-derived stem cells in fish collagen scaffolds for treatment of femoral head osteonecrosis

**DOI:** 10.1038/s41598-025-03015-6

**Published:** 2025-05-26

**Authors:** Pinxuan Zheng, Qi Jia, Zhongzhe Li, Heng Bo Jiang, Lu Zhou

**Affiliations:** 1 Department of Stomatology, The First Affiliated Hospital of Wenzhou Medical University, Wenzhou, Zhejiang China; 2 Department and Research Institute of Dental Biomaterials and Bioengineering, Yonsei University College of Dentistry, Seoul, Republic of Korea; 3 The CONVERSATIONALIST club & Department of Dental Digitalization, School of Stomatology, Shandong First Medical University, Jinan, Shandong China; 4 College of Sports Medicine and Rehabilitation, Shandong First Medical University & Shandong Academy of Medical Sciences, Tai’an, Shandong China

**Keywords:** Osteonecrosis of the femoral head, Marine biomaterials, Adipose-derived stem cells, Angiogenesis, Osteogenesis, Hippo pathway, Tissue engineering, Stem cells, Medical research

## Abstract

Osteonecrosis of the femoral head (ONFH) is a debilitating condition that often leads to femoral head collapse due to insufficient blood supply and impaired bone regeneration. However, effective treatment options for this condition are limited. This study explored a novel fish collagen (FC) scaffold combined with adipose-derived stem cells (ADSCs) to enhance osteogenesis and angiogenesis in ONFH. ADSCs were isolated and cultured on FC scaffolds to evaluate their biocompatibility and differentiation capacity. Osteogenic and angiogenic differentiation potentials were assessed in vitro, and the FC/ADSC combination was further evaluated in vivo using a rat model of ONFH. The molecular mechanisms were investigated via gene expression profiling and Hippo signaling pathway analysis. The FC scaffolds promoted ADSCs adhesion, proliferation, and migration without cytotoxicity. In vitro, FC/ADSCs significantly enhanced mineralization and capillary-like structure formation compared to the controls. FC/ADSCs improved bone regeneration and neovascularization in the femoral head in vivo, as confirmed by histological and immunohistochemical analyses. Mechanistically, the Hippo pathway is activated, increasing HIF-1α expression, which enhances osteogenic and angiogenic differentiation. FC scaffolds combined with ADSCs provide a promising therapeutic strategy for ONFH by facilitating bone regeneration and vascularization through the p-YAP/HIF-1α/VEGF axis. This scaffold-cell approach represents a potential advancement in ONFH treatment.

**Supplementary Information:**

The online version contains supplementary material available at 10.1038/s41598-025-03015-6.

## Introduction

Osteonecrosis of the femoral head (ONFH) is a prevalent condition that affects bone and joint function and is often triggered by trauma^1^, chronic alcohol consumption^2^, glucocorticoid use^3^, and osteoporosis^4^. The disease arises primarily from insufficient blood supply to the femoral head, which causes ischemia and leads to cell death^5^. However, the complete pathogenesis of OFHN remains unclear. The current understanding suggests that elevated intramedullary pressure disrupts the balance of osteogenic differentiation in stem cells and hinders angiogenesis. This compromised vascular regeneration and new bone formation significantly weakens the structural integrity of the femoral head, ultimately leading to its collapse^6,7^. OFHN is generally classified into conservative and surgical approaches^8^. Conservative approaches focus on reducing hip joint strain, medications, and rehabilitative exercises. Surgical approaches often yield better clinical and imaging results, making them the preferred interventions. Core decompression (CD) is a widely used surgical technique; however, it still faces challenges such as decreased postoperative mechanical strength and inconsistent outcomes, frequently culminating in femoral head collapse^9^. Various adjunctive therapies, including tantalum rod insertion, porous metal implantation, autologous bone grafting, and cell-based therapies, have been developed to address the limitations of CD^10–12^. Although these combinations have yielded varying results, they have also introduced new complications. Consequently, preserving the femoral head and its function while preventing collapse remains a pressing focus of the current research.

Porous tantalum rod (PTR) implantation has been widely used for early-stage ONFH patients, offering a potential delay or even prevention of total hip^13,14^. However, the long-term prognosis of PTR remains unsatisfactory^15^. In fact, a study by Ma et al.^16^suggested that PTR does not seem to be a viable option for treating ONFH. Therefore, the development of new implant materials is crucial. Biomedical materials such as PLGA (poly(lactic-co-glycolic acid)) have gained significant attention in scaffold design due to their biodegradability and biocompatibility, and it can effectively reduce the stress shielding. Ideal scaffold materials should synergize with seed cells with a good differentiation ability to produce good therapeutic effects. These cells can improve the regeneration of bone and blood vessels at the target location to ameliorate the degree of necrosis in the femoral head^17,18^. Recently, the use of osteoblasts and mesenchymal stem cells, such as adipose-derived stem cells (ADSCs), cultured and implanted in bone tissue engineering scaffolds, has emerged as a promising therapeutic approach for treating ONFH^19^. These treatments depend on the selection of seed cells and scaffold materials.

The scaffold acts as an extracellular matrix, providing an optimal microenvironment for cell growth^20^. High-performance scaffold materials that support the formation of vascularized bone tissue facilitate the rapid repair of defects in the femoral head. Wang et al.^21^ implanted composite scaffolds into an animal model of ONFH, resulting in substantial bone regeneration. Similarly, Zhang et al.^22^ used calcium phosphate-based composite PLGA microspheres in an ONFH animal model, which contributed to significant bone regeneration and vessel formation in the collapsed areas of the femoral head.

However, natural polymers have significant limitations, such as low mechanical strength and challenges in controlling degradation rates^23^. Synthetic polymers are also not ideal materials because of their rapid in vivo degradation and deformation^24^. Collagen, a natural biomaterial, has gained significant attention in recent years owing to its successful application in both soft^25^ and hard tissue repair^26^. Most commercial collagen products come from terrestrial animals, mainly bovine and porcine sources. However, mammalian collagen (MC) poses risks such as disease transmission like bovine spongiform encephalopathy and potential immune and inflammatory reactions^27^. In contrast, fish collagen (FC) offers superior biocompatibility, biodegradability, and lower immunogenicity, making it ideal for biomedical applications like tissue engineering scaffolds^28,29^. It is also cost-effective and free from religious restrictions, expanding its usability across diverse populations^30^. Elango et al.^31^ demonstrated that FC enhanced osteogenic differentiation without the use of additional induction agents. Bernhardt et al.^32^ showed that biphasic scaffolds composed of biomimetic mineralized salmon collagen and fibrillated jellyfish collagen, prepared via lyophilization and cross-linking, effectively supported osteogenic differentiation of human bone marrow mesenchymal stem cells (BMSCs). However, the denaturation temperature of fish collagen is related to their habitat. The disadvantage of most fish collagen is that its denaturation temperature is usually low, making it difficult to be used as an in vivo implant. The collagen derived from tilapia, a tropical fish, exhibits a denaturation temperature of approximately 37 °C, which enhances its potential as a substitute for mammalian collagen in biomedical applications^33^. Research indicates that tilapia collagen biomimetic materials are promising scaffolds for bone tissue engineering applications.

Early sources of seed cells were limited to chondrocytes^34^ and BMSCs^35^. Recently, adipose-derived stem cells (ADSCs) have emerged as viable options for bone repair and reconstruction. Compared to BMSCs, ADSCs are easier to obtain from adipose tissue using a simpler isolation process. Additionally, ADSCs exhibit strong proliferative and multidirectional differentiation potential^36^. Studies have shown that hydroxyapatite-coated porous titanium scaffolds coated with ADSCs improve bone regeneration and repair^37^. Jo et al.^38^ isolated and cultured autologous ADSCs from patients with osteoarthritis and injected them into joint cavities. After two years, the patient experienced significant pain relief and improved MRI scores. These findings suggest that ADSCs are promising seed cells that play a crucial role in bone defect repair. ADSCs for bone regeneration are expected to offer unique advantages and potential applications.

In this study, we combined FC artificial bone material with ADSCs to evaluate their biocompatibility and effects on the osteogenic and vascular differentiation of ADSCs. We also investigated the molecular mechanisms by which FC contributes to the treatment of ONFH. Based on these findings, we examined whether FC/ADSC implants could promote bone regeneration and neovascularization in bone defect areas using an ONFH animal model. This study aimed to provide a theoretical foundation for clinical interventions for ONFH and contribute to the advancement of treatment options for this condition.

## Materials and methods

### Preparation of ADSCs

For this study, ADSCs were harvested from the groin adipose tissue of 3–4-week-old rats. ADSCs were extracted using 0.1% Collagenase I and cultured. The surface antigens CD34, CD90, and CD105 were detected using a flow cytometer (BD Biosciences, USA). ADSCs were cultured in Osteogenic Differentiation Medium (high sugar DMEM cell culture medium containing 10% FBS + 100 nmol/L Dexamethasone + 50 µg/mL Ascorbic acid + 10 mmol/L β-Glycerophosphate) for 21 days. The cells were then stained with Alizarin Red S staining solution for 20 min, washed several times with ddH_2_O until clear, and observed for osteogenic differentiation using a microscope (Nikon, Japan). Matrigel matrix gel (50 µL) was added to pre-chilled 96-well plates and allowed to stand for 30 min. Cells were then seeded onto Matrigel-coated plates at a density of 8 × 10^4^ cells per well and incubated for 8 h. Angiogenic differentiation was observed under a microscope.

### Preparation of scaffolds

#### Preparation of the FC scaffolds

Tilapia skin were cut into thin slices (0.5 cm × 0.5 cm) and rinsed with distilled water to remove surface contaminants. The samples were then sequentially immersed in an 8% NaCl solution for 4 h to remove noncollagenous proteins and a 0.6% sodium dodecyl sulfate solution for 3 h to lyse the cells and release cellular contents. Subsequently, the samples were stirred at 60 r/min in a 5% n-butanol solution to extract lipids and reduce hydrophobic residue and a 0.2 M NaOH solution to eliminate residual non-collagenous proteins and further sterilize the material for 12 h each. The treated samples were then transferred to a 1 M acetic acid solution and stirred for 48 h to solubilize the collagen fibers, followed by the addition of pepsin and further stirring for another 48 h to improve its solubility and purity. Then the solution was centrifuged at 15,000 rpm for 20 min and following the microporous and multistage ultrafiltration membranes to remove impurities. The final product was a sterile, concentrated tilapia collagen with a molecular weight 100 kDa. The concentrated tilapia collagen was placed in a freeze-dryer for 72 h to obtain lyophilized tilapia FC sponges. The sponges were then sterilized using Co-60 irradiation and cut into blocks for further use.

#### Preparation of PLGA scaffolds

PLGA powder was dissolved in chloroform and mixed with NaCl at a ratio of 1:9. After thorough mixing, the prepared mold was filled, and 60 kg of pressure was applied for 24 h. They were then immersed in water to form porous scaffolds.

Electrophoretic analysis was performed to determine the protein purity of the scaffolds. The concentrated and separated gels were placed on an electrophoresis apparatus and injected with 1× electrophoresis buffer. The prepared samples were loaded into gel wells for electrophoresis. Electrophoresis was terminated when the sample reached approximately 1 cm from the lower end of the glass plate. The gel was then removed from the glass, fixed for 1 h, stained for 3 h, and decolorized until the protein bands appeared clearly on the polyacrylamide gel. Images of the stained gels were captured using an imaging device.

### Adhesion, proliferation, and migration of ADSCs on scaffolds

To evaluate the biocompatibility of the scaffolds, we assessed ADSC adhesion, proliferation, and migration. Cell adhesion determines whether the scaffold can effectively support cell attachment, which is a prerequisite for bone regeneration. Proliferation and migration assays further assess the cell viability of materials and ability of facilitating tissue integration. For the cells incorporation with scaffold, the prepared scaffolds were first rinsed twice with PBS, soaked in DMEM, and then transferred to 48-well plates. ADSCs in the logarithmic growth phase were seeded on one side of the sample surface at a density of 5 × 10^4^ cells. After incubation for 3 h, additional ADSCs were seeded on the opposite side of the material and incubated for another 3 h. Subsequently, 1 ml of culture medium was added to each sample, and incubation was continued. The FC/ADSCs were then gold-sputtered, and the adhension and condition of cells on the scaffolds were observed using scanning electron microscopy (SEM) to ensure the incorporation of ADSCs with scaffold. This incoraption method was also used for the histological evaluation in nude mice and animals modelling experiments.

#### Adhesion

ADSCs were seeded at a density of 5 × 10^4^ cells onto the surface of blank control, PLGA scaffolds, and FC scaffolds (*n* = 9). At 4-, 8-, and 12-h post-seeding, the scaffolds and adherent cells were incubated with MTT solution for 4 h at 37 °C. Subsequently, the scaffolds and adherent cells were washed three times with PBS and treated with DMSO for 10 min without light agitation. The absorbance was measured at 570 nm using an enzyme meter to quantify the ADSC adhesion ability. Each procedure was repeated three times.

#### Proliferation

ADSCs were seeded at a density of 5 × 10^4^ cells onto the surface of blank control, PLGA scaffolds, and FC scaffolds (*n* = 9). On days one, three, and five after cell seeding, the scaffolds and adherent cells were washed with PBS. The cells were then treated with MTT-containing medium for 4 h in direct contact with the scaffold samples. Following incubation, the cells were lysed with DMSO, and absorbance was measured at 570 nm using an enzyme meter to quantify the proliferative capacity of adipose stem cells. Each procedure was repeated thrice.

#### Preparation of conditioned medium (CM)

After the scaffolds were co-cultured with cells for 3 days, the supernatant was collected and centrifuged at 300 × *g* for 1 h to remove debris. Subsequently, the supernatant was filtered through 0.22 μm sterile microporous filters to obtain the CM under different groups. The CM was aliquoted and stored at -20 °C.

#### Migration

ADSCs (1 × 10^5^ cells/well) were suspended in 200 µL of DMEM without FBS and seeded into the upper chamber of Transwell inserts placed in a 24-well plate. In the lower chamber of the Transwell, 500 µL of conditioned medium containing release factors from the FC and PLGA scaffolds (with 10% FBS) was added separately. Another 500 µL of DMEM with 10% FBS served as the control medium group. After 24 h of incubation, the cells on the upper surface of the filter membrane were gently wiped with a cotton swab. Subsequently, the cells were fixed with 4% paraformaldehyde and stained with 1% crystal violet solution. Finally, the cells were observed under a microscope.

### Osteogenic and angiogenic properties of scaffolds with ADSCs

#### Young’s modulus

The Young’s moduli of the scaffold/cell constructs were examined on days 14 and 21 after osteogenic differentiation. The FC and PLGA scaffolds were cut into 10 × 10 × 10 mm cubes and sterilized. ADSCs were seeded onto the surface of the FC and PLGA scaffolds, respectively. Once the cell density reached 90% confluence, the culture medium was replaced with an osteogenic induction solution. On days 14 and 21 post-induction, the scaffold/cell constructs were evaluated using a compression test with an Instron universal testing machine. Before testing, the scaffold/cell constructs were washed twice with PBS. The compression speed was set to 5 mm/min, and the compression modulus was calculated.

#### Histological evaluation

To assess the osteogenic and angiogenic capabilites of the scaffolds in a controlled environment, we firstly conducted a subcutaneous implantation model in nude mice. Unlike the ONFH model, this system allowed to evaluate direct scaffold-cell interactions without the influence of bone remodelling mechanisms.

Osteogenic and angiogenic effects were examined in vivo over an 8-week period vivo. Twelve nude mice were randomly divided into two groups of six mice each. After anesthetizing the mice with 1% sodium pentobarbital, the surgical site was sterilized using alcohol swabs. An 8-mm incision was made between the shoulders of each mouse, and a 0.6 cm^2^ pocket was created on both sides of the incision to disrupt the fascia. Scaffolds with a diameter of 5 mm and thickness of 2 mm were implanted into the pockets: one side received scaffold/cell constructs, and the other side received FC scaffolds with ADSCs. After eight weeks, the mice were euthanized, and the implanted scaffolds were removed and fixed with 4% paraformaldehyde. Routine paraffin sections were prepared, followed by H&E staining and immunohistochemical staining (CD31, the endothelial cell marker protein). Histological evaluation was performed to observe new bone formation and neovascularization.

#### Western blots

To analyze the expression levels of proteins involved in bone formation, angiogenesis, and cell migration, proteins extracted from the tissues around the scaffolds in nude mice were subjected to western blot analysis. The tissue was rinsed with PBS, followed by extraction of total proteins using lysis buffer (RIPA, PMSF, and protease inhibitors). SDS-PAGE was performed to separate the proteins. After electrophoresis, the separated proteins were transferred onto PVDF membranes. Membranes were blocked with 5% skim milk and shaken gently for 2 h at room temperature. Primary antibodies specific to ALP, BMPR2, ANG-1, MMP-9, and β-actin, diluted in 5% skim milk, were then applied to the membranes and incubated overnight at 4 °C with slow shaking. After incubation, the membranes were washed thrice with wash buffer and subsequently incubated with secondary antibodies diluted in 5% skim milk on a shaker for 1 h at room temperature with gentle shaking. The entire membrane was uniformly covered with an ECL developer (China), and the imaging assay was conducted to visualize and analyze the expression levels of ALP, BMPR2, ANG-1, MMP-9, and β-actin using ImageJ software for semi-quantitative analysis.

### Screening of mechanisms and pathways related to FC-promoted differentiation of ADSCs

Given that ONFH is associated with disrupted osteogenesis and angiogenesis, understanding the gene regulatory networks and signaling pathways is crucial for elucidating how FC scaffolds promote bone and vascular regeneration. We retrieved the GSE74089 dataset, which is associated with femoral head necrosis, from the GEO database. To enhance the comprehensiveness of our analysis, we included the following additional datasets: GSE208310 (angiogenesis), GSE185874 (mesenchymal stem cell 3D culture), and GSE12021 (orthopedic disease). All data were sourced from public databases. We utilized GEO2R to analyze the raw gene expression data and identify differentially expressed genes (DEGs), applying the criteria |log2FC| > 0.5 and *p* < 0.05. A Venn diagram was used to visualize the overlapping DEGs across the datasets.

Functional and pathway enrichment analyses were conducted using the DAVID platform to explore the biological processes (BP), cellular components (CC), and molecular functions (MF) involved in treating femoral head necrosis using FC scaffolds. The analyses focused on GO and KEGG enrichment, with the top 10 KEGG pathways displayed in a bar chart. Additionally, the top ten items from the GO enrichment analysis were visualized using both bubble and bar charts, facilitated by the online platform Microbioinformatics, to identify potentially relevant signaling pathways and biological processes.

A protein-protein interaction (PPI) network was constructed using the STRING platform and subsequently imported into Cytoscape software for enhanced analysis. In Cytoscape, node colors were adjusted based on their degree values to emphasize key targets. The cytoHubba plug-in, integrated into the software, facilitated the identification and filtering of the top 10 hub target genes for further scrutiny.

### In vitro identification of the mechanisms and pathways

After the potential pathways involved in FC/ADSCs differentiation, the in vitro experiments were designed to experimentally validate these pathways. ADSCs were cultured in an oxoid anaerobic jar with an anaerobic gas pack to simulate the ischemic and hypoxic microenvironments induced by vascular injury during femoral head necrosis in vitro. The anaerobic pouch within the sealed jar rapidly absorbed atmospheric oxygen, generating CO_2_ and reducing the oxygen concentration to < 1% within an hour. The cells were divided into four groups: NC (no treatment), hypoxia (12 h of hypoxia), conditioned media + 12 h of hypoxia (FC/CM), and lysophosphatidic acid + 12 h of hypoxia (LPA).

#### Western blots

The preparation steps for CM were same as those described previously. The centrifuge was pre-cooled to 4 °C, and ice production was started in advance. Using a cell scraper, cells were carefully detached from the culture dish and transferred into a 1.5 mL centrifuge tube. A mixture of RIPA lysis buffer and phenylmethylsulfonyl fluoride (PMSF) was prepared at an appropriate ratio, added to the cell lysate, and allowed to lyse on ice for 30 min. Following lysis, the sample was centrifuged, and the protein concentration was measured. The Western blot procedure was conducted as previously outlined, with a specific focus on verifying the expression levels of HIF-1α and the Hippo pathway-related proteins YAP and p-YAP.

#### Angiogenic differentiation

Matrigel tube formation test was conducted to verify whether the scaffold can promote the differentiation of ADSCs into vascular endothelial cells. Matrigel was thawed at 4°C and aliquoted before the experiment. The essential experimental items were also pre-chilled in a 4°C refrigerator. At the start of the experiment, 50 µL Matrigel was added to each well of a pre-chilled 96-well plate to ensure that no bubbles were formed. The plates were then placed in a cell incubator for 60 min. Cells were seeded at a density of 8 × 10^4^ cells/well onto Matrigel-coated plates and incubated for 8 h. After incubation, the cells were observed under an optical microscope to check for the formation of tube-like structures.

#### Osteogenic differentiation

ADSCs were seeded in six-well plates at a density of 5 × 10^4^ cells per well. Once the cells reached 80% confluence, they were subjected to hypoxia treatment, followed by the addition of osteogenic differentiation medium containing 10 mM β-glycerophosphate, 100 nM dexamethasone, and 50 µg/mL ascorbic acid. After 21 days of inducing differentiation, Alizarin Red S staining was performed to assess the formation of calcified nodules. The cells were fixed with 4% paraformaldehyde at room temperature for 15 min, washed twice with PBS, and stained with 1 mL of Alizarin Red solution for 20 min. The staining solution was replaced with ddH_2_O, and the cells were washed several times until the background was clear. The stained matrices were observed and photographed under a microscope.

### Animals modelling

#### Animals

The animal study was carried out in accordance with the approved institutional guidelines and all involved methods were reviewed and approved by the Biomedical Research Ethics Committee of Shandong First Medical University (Shandong Academy of Medical Sciences, Shandong Province, China) under Approval NO. W202305110273. Male Sprague-Dawley (SD) rats, aged 7–8 weeks, provided by the Laboratory Animal Center of Shandong First Medical University, were selected for this study. The rats were divided into five groups: normal control (NC), ONFH, CD, PLGA/ADSCs, and FC/ADSCs. All methods were reported in accordance with ARRIVE guidelines (https://arriveguidelines.org).

#### ONFH modeling

After anesthetizing the rats with an injection of 3% sodium pentobarbital, their skin was disinfected with iodine and alcohol. Both lower limbs were fixed in an abducted position, while the rats were placed in a prone position. An incision was made on the lateral side of the greater trochanter of the femur, followed by separation of the muscle and soft tissues to expose the greater trochanter. The joint capsule was incised, and the femoral head was dislocated. The hip joint was reset to the level of the intertrochanteric line after stripping the supporting band. The wound was thoroughly flushed and sutured, and postoperative antibiotics were administered to prevent infection.

#### Groups

One month after the initial modeling, the rats were randomly divided into four groups. The ONFH group received no further treatment.

CD Group: After anesthesia, the rats were placed in the prone position, and the femoral head was re-exposed through the original surgical incision. A 1-mm-diameter surgical drill was used to create a bone tunnel from the greater trochanter to the femoral head, 2 mm below the cartilage. The necrotic bone was carefully removed from the femoral head while avoiding damage to the epiphyseal plate. The joint cavity was rinsed with saline to remove bone debris, and the wound was sutured and sterilized without any scaffolding material.

PLGA/ADSC Group: PLGA/ADSCs were implanted into the bone tunnels created as described above, lightly compacted, and the site was fully hemostatized and sutured sequentially.

FC/ADSC group: FC/ADSCs were implanted into the bone tunnels in the same manner as in the PLGA-ADSC group, lightly compacted, and the site was fully hemostatised and sutured sequentially. Postoperatively, the trauma and gait of the rats in each group were observed daily. Eight weeks after surgery, the rats were euthanized with pentobarbital sodium (150 mg/kg), and their femurs were immediately collected.

### Imaging, histological and molecular analysis

#### Micro-CT

Soft tissues were excised, and femurs were fixed for further analysis. Micro-computed tomography (micro-CT) was performed to examine the structure of the trabecular and cortical regions of the femur. After reconstruction using specialized software, three-dimensional images were analyzed, focusing on key parameters such as bone volume (BV), bone volume to total volume ratio (BV/TV), trabecular number (Tb.N), and trabecular separation (Tb.Sp).

#### Histological and immunohistochemical analyses

Sample Processing and Sectioning: Femoral tissue was decalcified in 10% EDTA for 4 weeks with twice-daily solution changes. The tissue was dehydrated in graded ethanol, cleared in xylene, embedded in paraffin, and sectioned at 5 μm thickness.

H&E Staining: Sections were deparaffinized, rehydrated, stained with hematoxylin and HE, and observed under a microscope.

Safranin O-Fast Green Staining: Sections were deparaffinized, rehydrated, stained with Fast Green for 5 min, differentiated in 95% ethanol, stained with Safranin O for 2 min, cleared in xylene, and mounted for microscopic observation.

Immunohistochemistry: Decalcified tissue sections were subjected to antigen retrieval, blocking, and staining with primary antibodies, VEGF (key factors that promote angiogenesis) and CD31 overnight at 4 °C. After secondary antibody application and DAB visualization, the sections were counterstained with hematoxylin, dehydrated, cleared, and mounted for microscopic observation.

Western Blot. After euthanizing the rats in each group, femoral head samples were collected, washed with PBS, and pulverized in liquid nitrogen. Total protein was extracted from the bone tissue using a pre-prepared lysis buffer. The Western blot experimental procedures, as detailed above, were followed to verify the expression levels of HIF-1α and Hippo pathway-related proteins YAP, p-YAP, and LATS1.

### Statistical analysis

Each experiment was repeated thrice. All data are presented as the mean ± standard deviation. Statistical analyses were performed using GraphPad Prism version 6.02 (GraphPad Software, San Diego, CA, USA). An independent sample t-test was used for comparisons between two sample means, whereas one-way ANOVA was used for comparisons among multiple groups. Statistical significance was set at *p* < 0.05.

## Results

### Cell morphology, surface markers, multidirectional differentiation potential

**Fig. 1:**
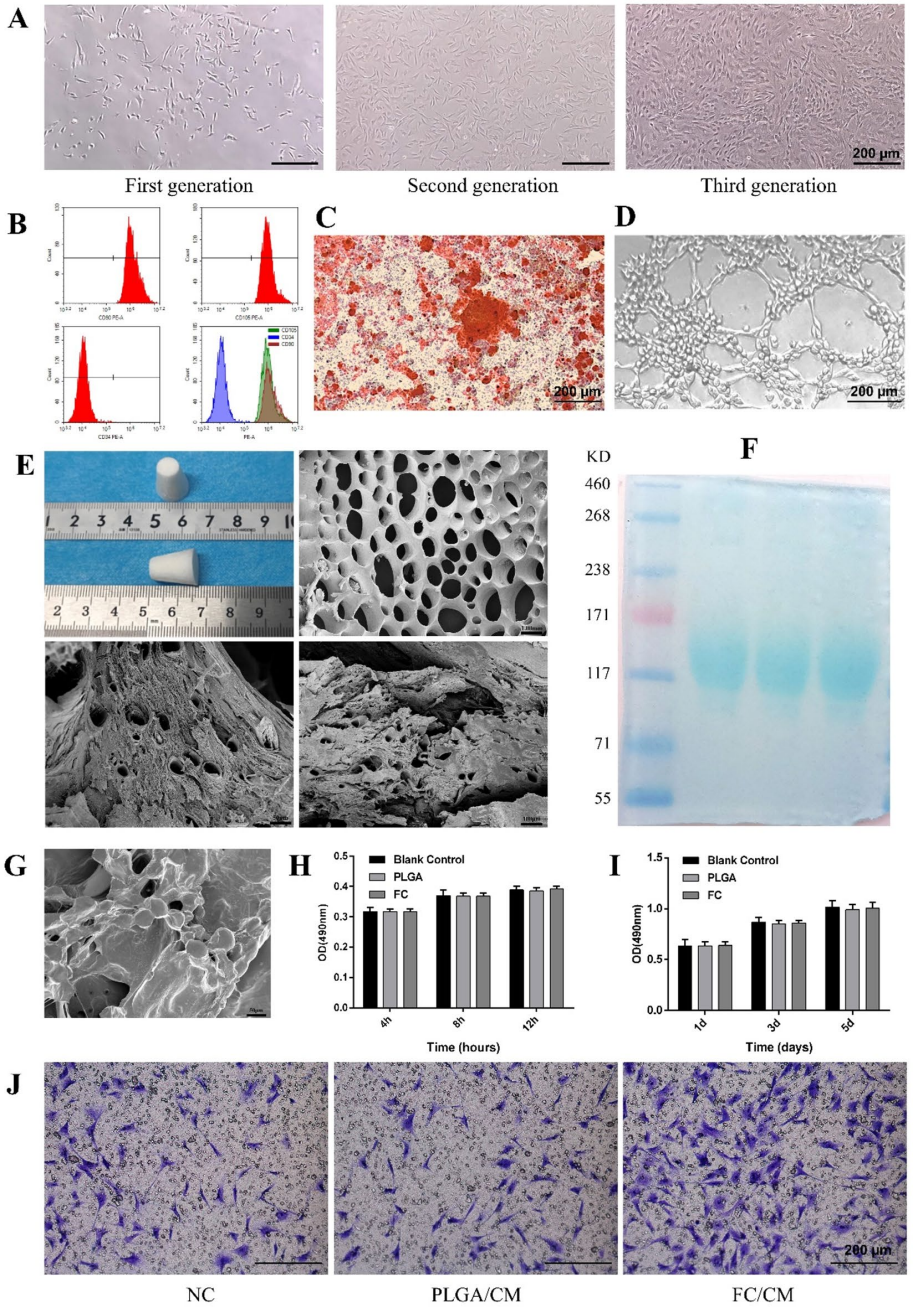
Characterization of FC and ADSCs. (**a**) Cell morphology of rat ADSCs. (**b**) Flow cytometry analysis of the expression of cell surface markers (CD34, CD90, and CD105). (**c**) Alizarin red staining of ADSCs after the induction of osteogenic differentiation. (**d**) Tube-like structures of ADSCs after the induction of angiogenic differentiation. (**e**) Porous morphology of the FC scaffold. (**f**) SDS-PAGE results for FC. (**g**) ADSCs adhesion on the FC surface. (**h**) Adhesion ability of ADSCs on the FC surface. (**i**) ADSCs’ proliferation on the FC surface. (**j**) ADSCs migrating through the chambers in the Transwell assay.

As Fig. 1(a) shows, the first-generation ADSCs exhibited various shapes, including short spindles and slender fiber-like forms. As the number of cell passages increased, the cell proliferation rate also increased. In the second generation, ADSCs displayed a uniform shuttle-shaped morphology similar to that of adherent cells. Third-generation ADSCs consistently exhibited a fibroblast-like long spindle shape.

Surface marker analysis revealed that ADSCs expressed CD90 and CD105, which are characteristic markers of mesenchymal stem cells (MSCs), with minimal expression of CD34 (Fig. 1(b)). These results confirmed that the ADSCs isolated in this study possessed the typical morphology and surface characteristics of MSCs.

After 21 days of culture, ADSCs were positively stained with Alizarin Red S, as evidenced by the formation of mineralized nodules (Fig. 1(c)). This positive staining suggests that the extracted ADSCs had the potential to differentiate into osteoblasts. In vitro angiogenesis experiments demonstrated that when ADSCs were seeded on the surface of a Matrigel matrix gel and incubated for 8 h, they formed tube-like structures (Fig. 1(d)), indicating the potential for ADSCs to differentiate into endothelial cells. These findings support the multidirectional differentiation potential of ADSCs.

### Characteristics of FC

We verified the surface characteristics and structure of the FC (Fig. 1(e)). It appeared milky white, porous, and fluffy. Scanning electron microscopy revealed a regular porous honeycomb structure with an impurity-free, smooth surface. Additionally, the collagen SDS-PAGE results for FC (Fig. 1(f)) showed clear protein bands without diffuse or trailing bands, demonstrating its high purity.

### ADSCs attachment, proliferation, and migration

After co-culture, the cells adhered well to both the surface and the voids of the FC scaffolds, as observed under an electron microscope. The cells on the FC/ADSCs were in good condition, indicating that the FC scaffold was biocompatible and exhibited no significant cytotoxicity (Fig. 1(g)). After observing cell attachment under a microscope, further quantitative analysis was performed on FC/ADSCs, with PLGA/ADSCs serving as the control group. The MTT assay results (Fig. 1(h)) showed no significant differences in absorbance between the FC/ADSCs, PLGA/ADSCs, and blank groups. This indicated that the scaffolds did not exhibit cytotoxicity towards ADSCs. In addition, the optical density (OD) of each scaffold increased over time, suggesting successful normal cell adhesion.

Absorbance at 1 day, 3 days, 5 days was measured to assess the proliferation of ADSCs on the scaffolds (Fig. 1(i)). The absorbance of both scaffold types increased over time and was not significantly different from that of the blank group. This indicated that cell proliferation on the scaffolds was normal.

The effect of FC on the migration ability of ADSCs was assessed using a transwell assay. No significant difference was observed in the PLGA/CM group compared to the control group, whereas the FC/CM group exhibited a higher chemoattractive potential, with a significant increase in the number of cells crossing the chambers (Fig. 1(j)). This suggests that FC promoted ADSCs migration.

### Osteogenicity and angiogenicity of scaffolds/adscs

**Fig. 2:**
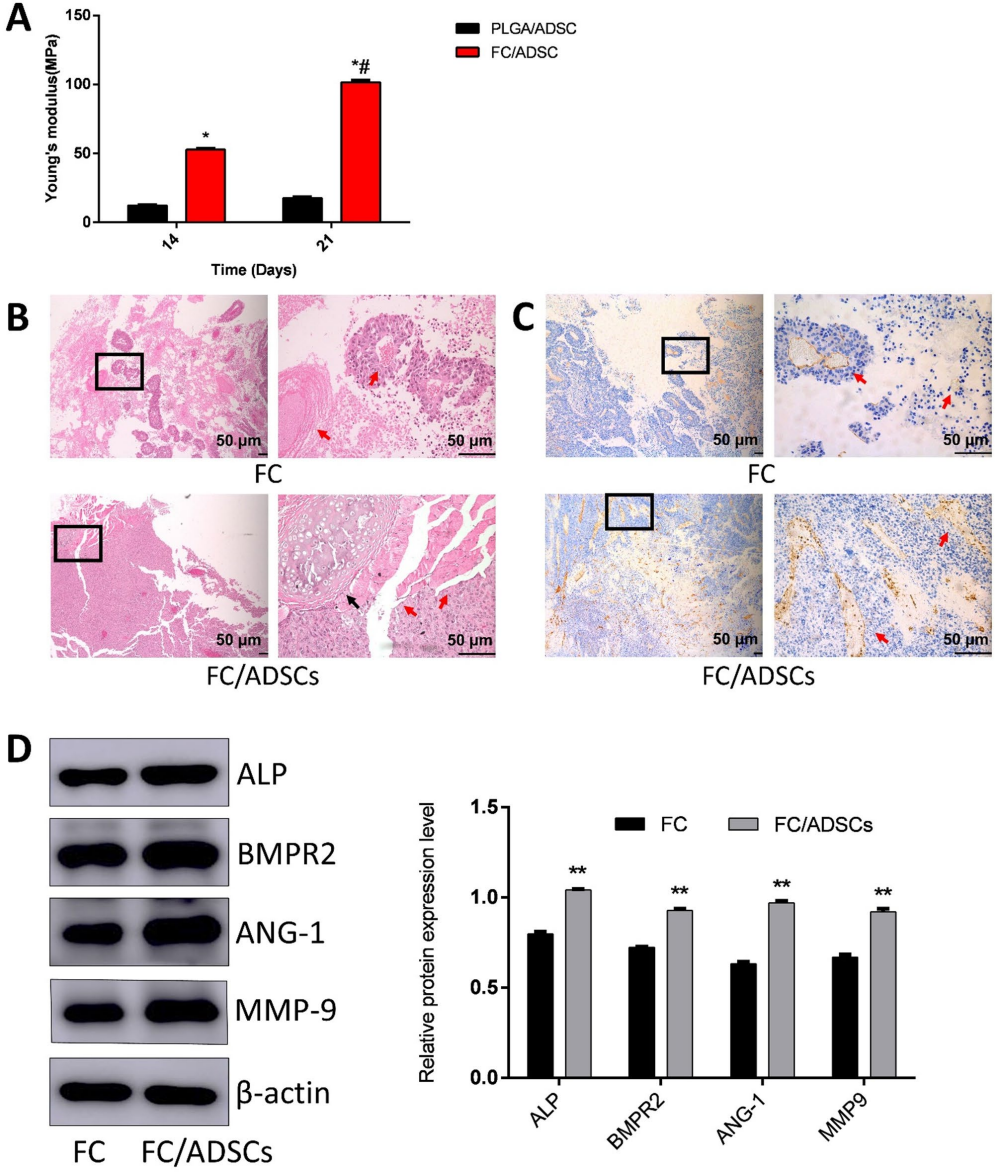
FC enhanced osteogenic and angiogenic differentiation of ADSCs. (**a**) Mechanical Properties of Scaffold/ADSCs after 14 and 21 days of osteogenic-induced differentiation. * *p* < 0.05, compared to the PLGA/ADSC group on the same day; # *p* < 0.05, compared to the PLGA/ADSC group on 14 days. (**b**) H&E staining of the tissue around the scaffolds in pockets of nude mice. In the FC group, the areas indicated by red arrows represent loose fibrous tissue structures. In the FC/ADSCs group, the red arrows indicate dense ectopic bone tissue stained in red. The black arrow denotes vascular endothelial-like structures. (**c**) Immunohistochemical staining for CD31 in the tissue around scaffolds in the pockets of nude mice. (**d**) Western blotting results of the tissue around scaffolds in pockets of nude mice. * *P* < 0.005 compared to the FC group.

The osteogenic properties of the scaffold/ADSCs were evaluated by measuring their Young’s moduli after 14 and 21 days of osteoinductive differentiation. As shown in Fig. 2(a), Young’s modulus of 21-day FC/ADSCs (101.52 ± 1.81) was approximately six times higher than that of 14-day FC/ADSCs (17.69 ± 0.85), indicating increased calcium nodule formation over time. Additionally, the Young’s modulus of the FC/ADSCs was approximately five times higher than that of the PLGA-ADSC complex. These results demonstrate that FC/ADSCs exhibited superior osteogenic effects.

The nude mice used to assess angiogenic and osteogenic effects in vivo showed no mortality or good wound healing throughout the test period. H&E staining (Fig. 2(b)) revealed that the FC group without ADSCs exhibited little neovascularization and loose fibrous tissue with almost no osteoid cells present. In contrast, significant angiogenesis, with more vascular endothelial-like structures and ectopic bone tissue formation, was observed in the FC/ADSC group. In addition, the FC/ADSC group displayed uniform cell dispersion, which was attributed to the micron-sized honeycomb pores in the scaffolds that facilitated cell migration. This suggests that FC promotes angiogenesis and induces ectopic bone formation by enhancing the migration and differentiation of ADSCs. Immunohistochemical staining for CD31 in the scaffold tissue of each group further supported these findings. Strong brown staining of the walls of the tube-like structures, indicated by the red arrow in Fig. 2(c), confirmed the presence of CD31 and neovascularization. Immunostaining in the FC/ADSC group was significantly stronger than that in the FC group, indicating greater formation of tube-like structures in the FC/ADSC group.

Western blotting results of proteins from the tissues are shown in Fig. 2(d). The expression levels of the representative bone formation marker proteins, ALP and BMPR2, were significantly higher in the FC/ADSC group than in the FC group. Additionally, the relative expression of the angiogenesis marker protein ANG-1 was significantly increased in the FC/ADSC group. As collagen, the main component of FC, promotes cell migration, we examined the expression of pro-migratory proteins. The results showed that the expression level of MMP-9 was higher in the FC/ADSC group than in the FC group. This indicates that FC/ADSCs can enhance cell migration and differentiation. These findings are consistent with the results of in vitro transwell assays and osteogenic and angiogenic-induced differentiation studies.

### Mechanisms and pathways related to FC-promoted differentiation of ADSCs

**Fig. 3:**
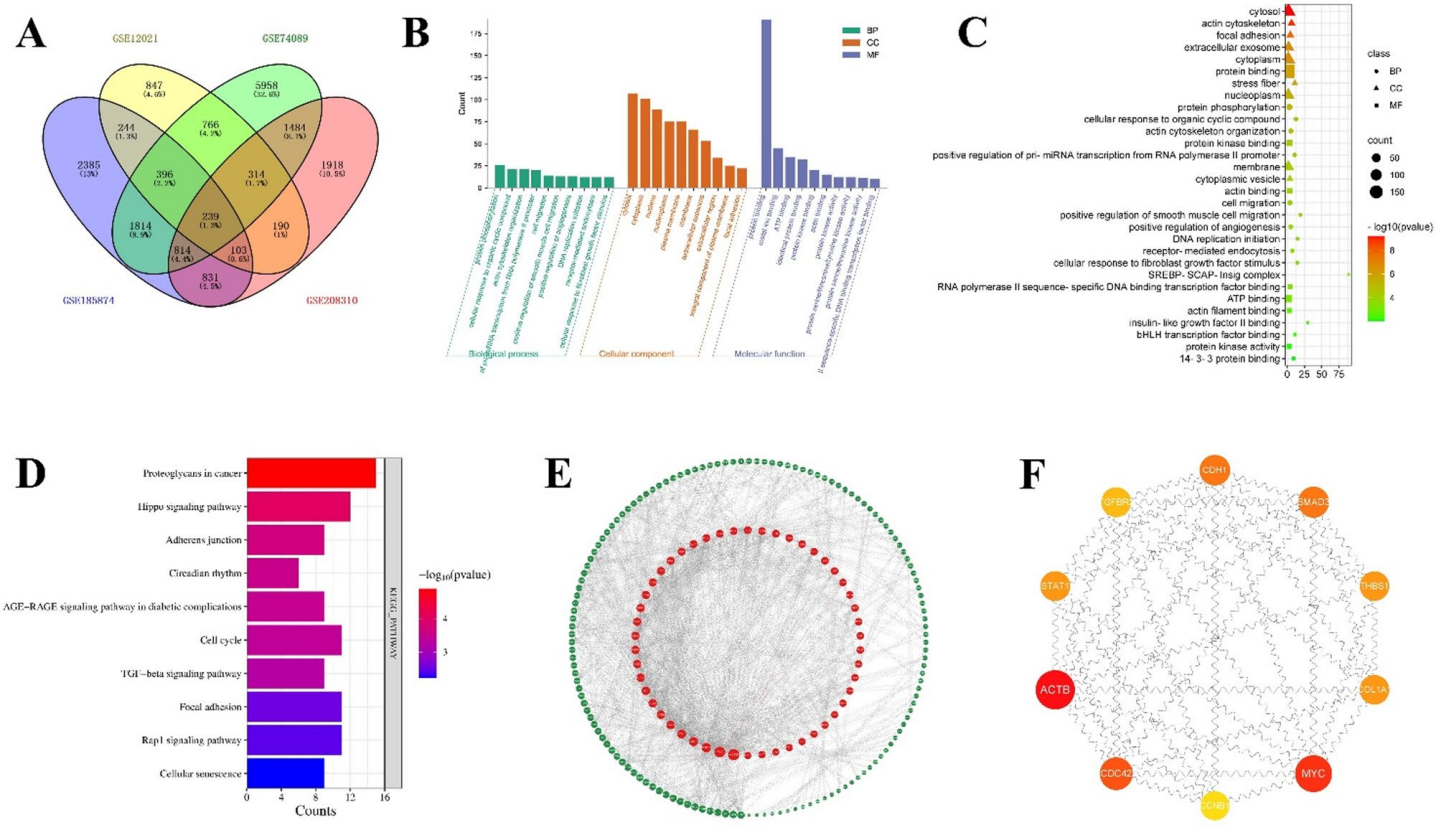
Screening and identification of mechanistic pathways related to FC promotion of angiogenic differentiation in ADSCs. (**a**) Venn diagrams for four GEO datasets (GSE12021, GSE74089, GSE185874 and GSE208310). (**b**) Top 10 genes from GO enrichment analysis in biological process (BP), cellular component (CC), and molecular function (MF). (**c**) GO enrichment analysis. A higher count suggested a greater number of gene associations. Red indicates a lower p-value, meaning higher significance, while green indicates a higher p-value, meaning lower significance. (**d**) Top 10 pathways from KEGG enrichment analysis. Red indicates a lower p-value, meaning higher significance, while blue indicates a higher p-value, meaning lower significance. (**e**) Construction of the PPI network. (**f**) Hub genes were obtained using Cytoscape.

To investigate the molecular mechanisms of FC scaffolding in the treatment of femoral head necrosis, we searched the GEO database for relevant datasets. Based on preliminary findings suggesting its role in promoting angiogenesis, we included additional datasets related to angiogenesis. By intersecting the filtered GSE12021, GSE74089, GSE185874, and GSE208310 datasets, 239 differentially expressed genes were identified (Fig. 3(a)). The top 10 genes from the GO enrichment analysis were then visualized, as shown in Fig. 3(b-c). The results showed that the enriched biological processes included protein phosphorylation, cell migration, and positive regulation of angiogenesis. The cellular components highlighted were the cytoplasm, actin cytoskeleton, and focal adhesions, whereas the main molecular functions were protein binding and protein kinase binding.

To investigate the signaling pathways potentially involved in the treatment of femoral head necrosis with the FC scaffold, we conducted a KEGG enrichment analysis. This analysis identified 41 relevant signaling pathways, with 10 pathways selected based on the criterion of *p* < 0.05 (Fig. 3(d)). The key differentially expressed genes were primarily enriched in pathways such as proteoglycans in cancer, the Hippo signaling pathway, and adherens junctions.

Additionally, a protein-protein interaction (PPI) network was constructed using Cytoscape software, as shown in Fig. 3(e). In this network, nodes with larger sizes and deeper red colors represent more important genes. The top 10 hub target genes were identified using the CytoHubba plug-in, as shown in Fig. 3(f), with the key genes including ACTB, COL1A1, STAT1, and THBS1.

### FC promotes HIF-1α expression by activating the Hippo signaling pathway

**Fig. 4:**
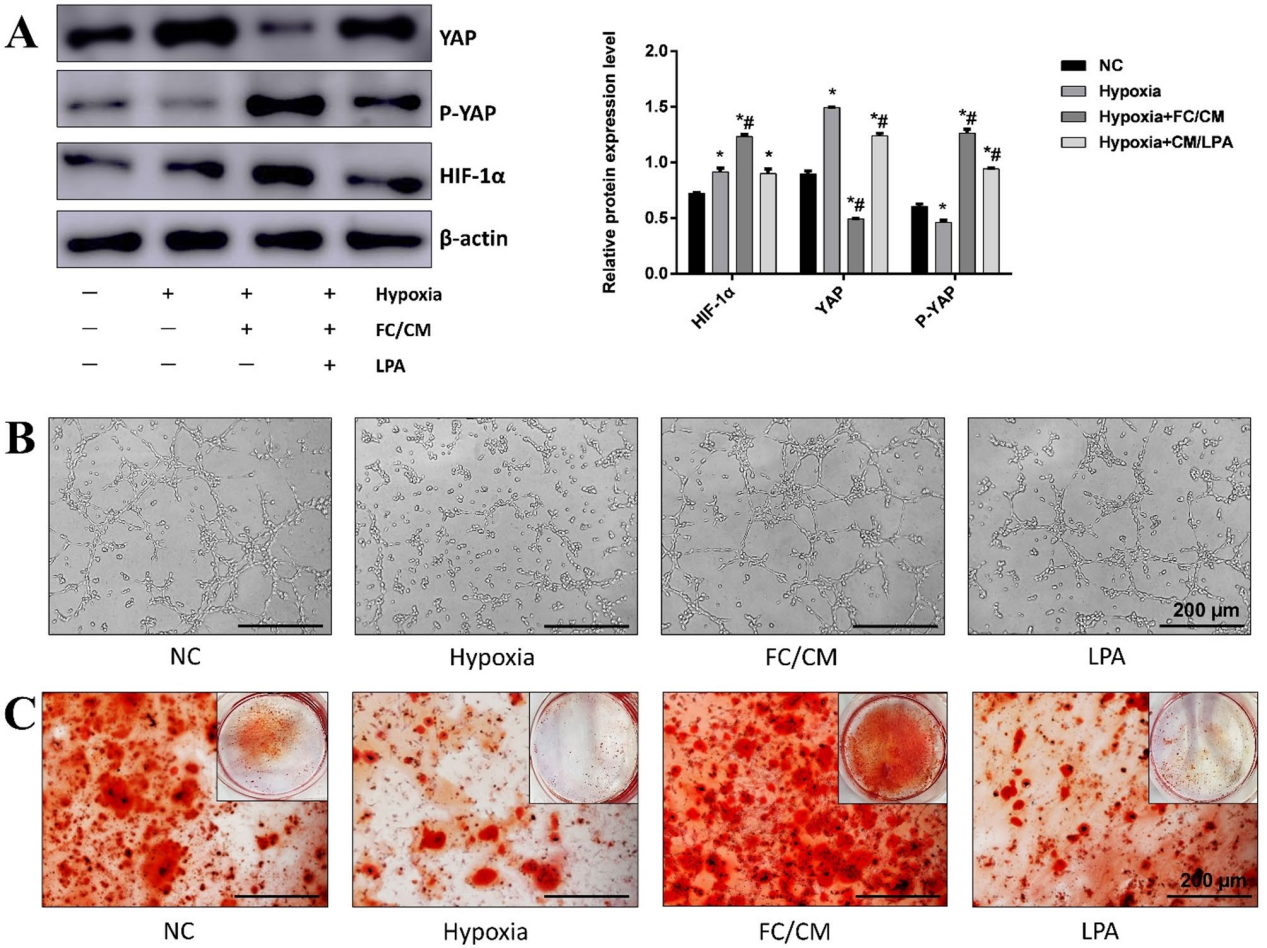
FC promotes angiogenic and osteogenic differentiation of ADSCs by activating the Hippo pathway. (**a**) Relative expression of YAP, p-YAP, and HIF-1α proteins. (**b**) Tube formation of ADSCs on Matrigel after 8 h. (**c**) Alizarin Red staining of ADSCs after 21 days of osteogenic-induced differentiation.

KEGG enrichment analysis indicated that the Hippo signaling pathway may be a key target for FC scaffolds in the treatment of ONFH. To validate this bioinformatics prediction, we conducted western blot experiments, with the results presented in Fig. 4(a). First, we exposed ADSCs to a hypoxic environment in vitro to mimic the vascular injury conditions characteristic of ONFH. YAP expression increased, and p-YAP expression decreased compared to those in the control group. When CM was added under hypoxic conditions, the opposite was observed, with increased p-YAP expression and decreased YAP expression. This suggests that FC can activate the Hippo signaling pathway, thereby promoting downstream target expression. To determine whether HIF-1α plays a crucial role in angiogenesis, we measured its expression levels. In the FC group, HIF-1α expression was significantly increased compared to that in the hypoxia group.

To further explore the relationship between HIF-1α elevation and Hippo pathway activation, we stimulated YAP expression using lysophosphatidic acid (LPA). The results showed that HIF-1α expression was suppressed, and p-YAP expression was downregulated in cells treated with LPA under CM conditions. This indicates that FC promotes the phosphorylation of YAP, keeping it in the cytoplasm where it is degraded, preventing its nuclear entry, and influencing HIF-1α expression. These findings suggest that FC scaffolds may exert therapeutic effects on ONFH via the p-YAP/HIF-1α/VEGF axis.

To evaluate the ability of ADSCs to promote vascularization in vitro, we inoculated the cells onto the surface of the matrix gel and observed the formation of a three-dimensional mesh-like structure resembling a vascular lumen, as shown in Fig. 4(b). Complete tube-like structures were formed in the control group, whereas the hypoxic group exhibited incomplete structures. The FC group generated a significantly higher number of angioid structures than the hypoxic group. However, after the addition of LPA, the formation of these structures was incomplete, which aligns with the western blot results.

The osteogenic differentiation potential of ADSCs was assessed by observing the formation of mineralized nodules following osteogenic induction (Fig. 4(c)). The hypoxic group had fewer mineralized nodules, as indicated by the reduced positive Alizarin Red S staining, compared to the control group. In contrast, the FC group showed a significant increase in the number of mineralized nodules. However, after the addition of LPA, the number of mineralized nodules decreased. These findings further confirm that FC promotes osteogenic differentiation of ADSCs and enhances their mineralization activity.

### Femoral head micro-CT analysis

**Fig. 5:**
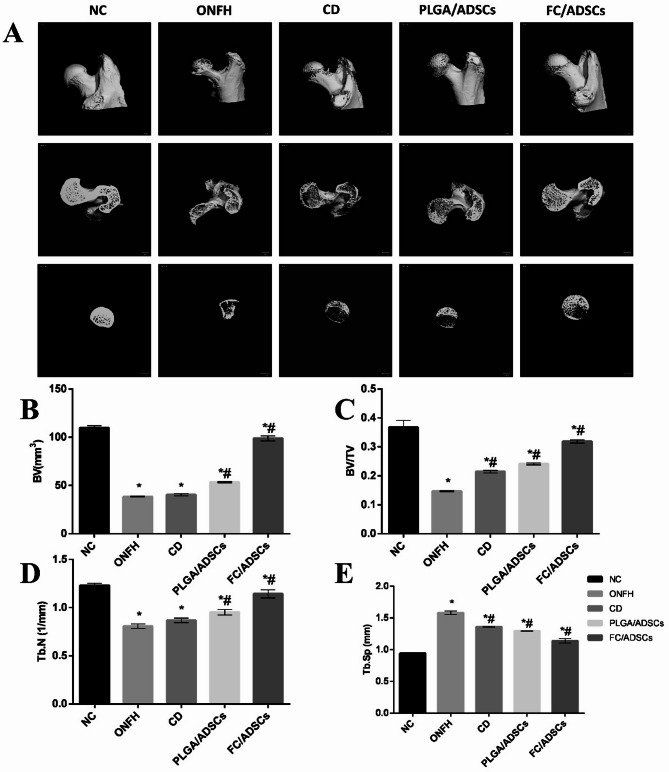
Micro-CT analysis of the femoral head. (**a**) Micro-CT images of the femoral head. (**b**–**e**) Bone trabecular parameters, including BV, BV/TV, Tb.N, and Tb.Sp. * *p* < 0.05, compared with the NC group; # *p* < 0.05, compared with the ONFH group.

As shown in Fig. 5(a), micro-CT scans revealed high bone mineral density (BMD) and no defects in the femoral heads of the control group. In contrast, the ONFH group exhibited osteoporosis, reduced bone mineral density (BMD), and significant defects that were partially alleviated in the CD group. Partial defect repair was observed in the PLGA-ADSC group, although a small amount of osteonecrosis persisted. The FC/ADSC group showed increased BMD, fewer defects, and almost no remaining osteonecrosis.

Data analysis from the micro-CT 3D reconstruction, shown in Fig. 5(b-e), indicated significant reductions in bone volume (BV), bone volume to total volume ratio (BV/TV), and trabecular number (Tb.N) in the ONFH group compared to the control group. These parameters improved in both the PLGA/ADSC and FC/ADSC groups compared to those in the ONFH group, with a more pronounced improvement in the FC/ADSC group. Additionally, trabecular separation (Tb.Sp) was significantly decreased in both the PLGA/ADSC and FC/ADSC groups compared to that in the control group. These results suggest that FC/ADSCs are effective in repairing femoral head defects in vivo.

### Histological and immunohistochemical analysis of the femoral head

**Fig. 6:**
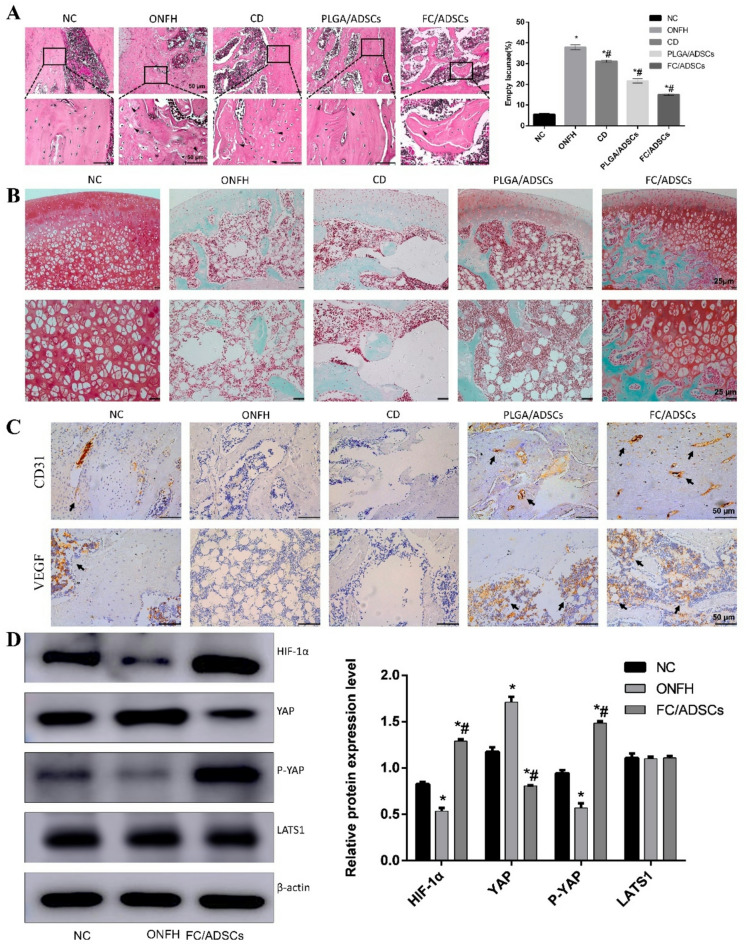
FC promoted angiogenic and osteogenic differentiation of ADSCs in ONFH rats after 8 weeks. (**a**) H&E staining of the femoral head with the proportion of empty lacunae in the femoral head in each group. (**b**) Safranin O-fast green staining of the femoral head. (**c**) Immunohistochemical staining (VEGF, CD31) of the femoral head. The black arrows indicate positively stained tube-like structures. (**d**) E Relative expression of HIF-1α, YAP, p-YAP, and LATS1. * *p* < 0.05, compared with the NC group; # *p* < 0.05, compared with the ONFH group.

Figure 6(a) displays the results of H&E staining, showing the number of osteocyte lacunae within the femoral heads of each group. The control group exhibited no obvious osteocyte lacunae, whereas the ONFH and CD groups showed large numbers. In the PLGA/ADSC group, the presence of osteocyte lacunae was reduced, whereas the FC/ADSC group had almost no osteocyte lacunae, indicating notable repair of femoral head necrosis.

Figure 6(b) displays Safranin O-fast green staining to further examine the structure of the articular cartilage and subchondral bone tissue. The ONFH and CD groups exhibited rough articular cartilage surfaces, sparse bone arrangement, uneven staining, and numerous destained osteoblasts. Conversely, the FC/ADSC group had a relatively thicker cartilage layer, more orderly bone arrangement, and uniform staining, confirming its effectiveness in treating ONFH.

We further investigated angiogenesis in the femoral head using immunohistochemical staining for CD31 and VEGF (Fig. 6(c)). In the ONFH and CD groups, CD31 and VEGF expression levels were significantly reduced, and the microvascular structure was damaged compared to the control group. The PLGA/ADSC group showed an increase in the number of CD31- and VEGF-positive cells with a higher vascular density. Notably, the FC/ADSC group exhibited the highest number of CD31- and VEGF-positive cells, increased vascular density, and a largely intact microvascular structure compared to the other groups.

### FC/ADSCs activate the Hippo pathway to enhance angiogenesis

The therapeutic mechanism of FC scaffolds was confirmed with the help of in vitro experiments. To further investigate the molecular mechanism of FC/ADSC treatment in rats with ONFH, we conducted in vivo western blot analysis, as shown in Fig. 6(d). Specifically, the HIF-1α protein level was reduced in the ONFH group compared to the control group, while the FC/ADSC group showed an increase in HIF-1α expression. These results indicate that FC/ADSCs decreased YAP expression and increased p-YAP expression without upregulating LATS1, an upstream target of p-YAP. This suggests that FC/ADSCs exert their therapeutic effects by regulating YAP phosphorylation. These findings suggest that FC/ADSCs may activate the Hippo signaling pathway, leading to the upregulation of HIF-1α. This, in turn, promotes the repair of bone defects by enhancing angiogenesis.

## Discussion

Stem cells are widely used as seed cells in bone tissue engineering. Stem cell therapy has been widely studied in the preclinical phase and is used clinically for tissue regeneration^39^. ADSCs have recently been widely studied owing to their easy isolation and good differentiation capabilities^40^. In this study, we extracted rat ADSCs and characterized them based on cell morphology, multidirectional differentiation potential, and surface markers. ADSCs displayed a spindle-like shape, which is typical of stem cells in vitro. Surface marker analysis showed high positivity for CD90 and CD105 and negativity for CD34, confirming typical mesenchymal stem cell (MSC) characterization. These results indicate that the isolated cells were high-purity ADSCs, meeting the experimental requirements of the study. ADSCs have demonstrated potential for osteogenic, angiogenic, and adipogenic differentiation. In this study, we focused on their osteogenic and angiogenic capabilities, as verified by Alizarin Red S staining and tubule formation assays. These analyses confirmed the osteogenic and angiogenic differentiation potential of the cells, which was consistent with the objectives of this study.

High-quality scaffolds provide an optimal environment for seed cell proliferation and differentiation. PLGA is a biodegradable polymer that is widely used in biomedical applications^41,42^. Previous research has shown that PLGA combined with ADSCs effectively repairs tibial defects^43^. In this study, we used a PLGA scaffold as a control to evaluate the biocompatibility and biological efficacy of FC. Collagen, an extracellular matrix component, promotes cell migration into defective areas while offering structural support. Collagen-based scaffolds, particularly those derived from marine sources, are increasingly being used in bone tissue engineering^44^. Studies have shown that marine collagen independently stimulates osteogenesis and promotes MSC activity^31^. In this study, the FC scaffold was bionically mineralized in vitro, creating a three-dimensional microenvironment and providing a stable matrix for cell proliferation^29^. It was observed under SEM as a regular porous honeycomb structure with a smooth, impurity-free surface. This porous structure facilitated cell migration within the scaffold^45^. The cells adhered well to the surface and displayed healthy growth. SDS-PAGE analysis confirmed the high purity of collagen. Cell adhesion and proliferation were assessed using the MTT assay, which indicated that FC was non-toxic to ADSCs and did not interfere with cell adhesion or proliferation. Scaffolds provide a conducive growth environment for cells, enabling the secretion of growth factors, cytokines, and other bioactive molecules. Cell migration was tested using CM. While the PLGA group showed no significant difference compared to the control group, the FC group exhibited a notably higher number of cells that migrated through the chambers, indicating that FC effectively enhanced cell migration. Therefore, we speculated that the release of collagen from the scaffold into the culture medium may have contributed to the enhanced vascularization. We have to report that the specific composition of the CM released during the co-culture period is not characterized. Although the enhanced angiogenic and osteogenic effects were observed, the exact molecular components responsible for these effects remain to be elucidated and will be the focus of future investigations.

Scaffolds are crucial for triggering cell differentiation and mineralization, which affects their mechanical properties^46^. Young’s modulus results indicated that FC promoted the osteogenic differentiation of cells more effectively. The FC/ADSC group exhibited more tube-like structures and bone tissue than the FC group, with cells uniformly distributed in the H&E staining results. Additionally, immunohistochemical staining revealed stronger CD31-positive expression in the FC/ADSC group. These findings suggest that FC scaffolds provide an enhanced microenvironment for the differentiation, angiogenesis, and osteogenesis of ADSCs.

Alkaline phosphatase (ALP) serves as a marker of osteoblast differentiation and bone mineralization^47^. BMPR2, a bone morphogenetic protein receptor, plays a critical role in bone development and morphology regulation, as well as in promoting angiogenesis and inhibiting adipogenesis^48^. ANG-1 is involved in essential processes, such as vascular endothelial cell proliferation, migration, and angiogenesis^49^. MMP-9 is a key inducer of angiogenesis and plays a significant role in tissue matrix degradation and cell migration^50^. In our study, we analyzed the expression of these proteins in samples implanted subcutaneously into nude mice and found that FC/ADSCs elevated the levels of ALP, BMPR2, ANG-1, and MMP-9. These results suggest that the FC scaffolds enhanced the proliferation and migration of ADSCs and supported osteogenic and angiogenic differentiation.

In the in vivo experiments, significant neovascularization was observed in the FC/ADSC group. To further investigate the underlying molecular mechanisms, we conducted a biosignal analysis. Our findings revealed that several related genes were enriched in the Hippo signaling pathway, suggesting that this pathway may play a role in the treatment of ONFH through FC scaffolds. The Hippo pathway involves a series of conserved kinases that are crucial for cellular differentiation^51^ and tissue regeneration^52^. Yes-associated protein (YAP), the primary effector of the Hippo pathway, is phosphorylated and subsequently degraded when the pathway is activated^53^. The activation of YAP can promote angiogenesis^54^ whereas inhibition of YAP expression has been shown to enhance angiogenesis in another study^55^. Additionally, Kuang et al.^56^ showed that some exosomes can effectively inhibit the progression of ONFH through the Hippo pathway. These findings suggest a close connection between the Hippo pathway and ONFH, potentially offering insights into therapeutic strategies for ONFH.

Ischemia^57^ and hypoxia^58^ are key factors in the progression of ONFH. Under hypoxic conditions, HIF-1α overexpression can enhance the osteogenic and angiogenic differentiation of stem cells^59^, and it can promote vascular repair and regeneration by stimulating VEGF expression^60^. There is evidence that YAP can bind with HIF-1α, potentially inhibiting HIF-1α activity and limiting the expression of vascular growth-associated endothelial genes^61^. These findings suggest that the Hippo pathway may regulate angiogenesis with HIF-1α. LPA, a YAP activator known to inhibit LATS1 and promote YAP activation both in vitro and in vivo^62^ was used to explore this mechanism. YAP expression was higher in the hypoxic group; YAP expression increased in the control group, whereas p-YAP expression decreased. However, after the addition of CM, these results were reversed, indicating that FC influences the Hippo signaling pathway. Additionally, the expression of HIF-1α was significantly elevated in the FC group. When LPA was applied in the presence of CM, we observed a significant inhibition of YAP expression. Incomplete tube-like structures were observed in the hypoxic group. In contrast, the FC group displayed a greater number of tube-like structures. Additionally, even with the addition of LAP, the LAP group still showed tube-like structures compared to the hypoxia group. Similar results were observed with Alizarin Red S staining. These findings indicate that FC provides a supportive microenvironment for both vascularization and osteogenesis of ADSCs. These results suggest that FC may promote YAP phosphorylation, thereby keeping YAP in the cytoplasm for degradation, rather than allowing it to enter the nucleus and inhibit HIF-1α.

In the early stages of ONFH, impaired microcirculation triggers new bone formation^22,63^. However, empty lacunae develop in later stages with a severely diminished capacity for vascular regeneration and new bone formation^64^. To more accurately simulate ONFH pathology, we established a traumatic ONFH model and conducted in vivo experiments^65^. Micro-CT imaging revealed that the bone reparative efficiency in the FC/ADSC group was much higher than that in the PLGA/ADSC group. H&E and safranin O/fast green staining further showed that FC/ADSCs reduced the number of empty lacunae in the femoral head and promoted osteogenic differentiation.

Angiogenesis is crucial for bone repair because bone microcirculation and osteogenesis are interdependent processes^66^. In the ONFH group, reduced CD31 and VEGF expression and significant disruption of tube-like structures were observed. In contrast, the FC/ADSC combination promoted vascular differentiation. We observed increased expression of p-YAP and HIF-1α, along with decreased YAP expression in the FC/ADSC group. This indicates that FC can activate the Hippo pathway and enhance HIF-1α expression. Notably, LATS1, an upstream regulator of p-YAP, was not upregulated in this group, suggesting that FC scaffolds may directly promote YAP phosphorylation.

Thus, FC/ADSCs effectively supported vascularization within tissue-engineered constructs, allowing nutrient transport, which is essential for bone regeneration. This enhances graft survival in vivo and ultimately improves the bone repair efficacy of the material.

## Conclusion

In this study, FC/ADSCs significantly enhanced osteogenesis and angiogenesis in patients with ONFH. The FC scaffolds provide a biocompatible microenvironment that supports ADSCs adhesion, proliferation, and differentiation. Mechanistically, we found that FC/ADSCs prompted activation of the Hippo signaling pathway to increase YAP phosphorylation and upregulate HIF-1α. Our findings demonstrate the therapeutic potential of combining ADSCs with FC scaffolds for ONFH treatment.

## Electronic supplementary material

Below is the link to the electronic supplementary material.

Supplementary Material 1

## Data Availability

All data supporting the findings of this study are included in the article.
