# Supplementary material for: Enhanced osteogenic and angiogenic capabilities of adipose-derived stem cells in fish collagen scaffolds for treatment of femoral head osteonecrosis
Source: Sci Rep. 2025 May 26;15:18300. doi: 10.1038/s41598-025-03015-6 (PMC12106632; doi:10.1038/s41598-025-03015-6)

Enhanced osteogenic and angiogenic capabilities of adipose-derived stem cells in fish collagen scaffolds for treatment of femoral head osteonecrosis

Pinxuan Zheng[1,#], Qi Jia[2,3,#], Zhongzhe Li[4,#], Heng Bo Jiang[3,*], Lu Zhou[4,*]

[1] Department of Stomatology, The First Affiliated Hospital, Wenzhou Medical University, Wenzhou, Zhejiang, China

[2] Department and Research Institute of Dental Biomaterials and Bioengineering, Yonsei University College of Dentistry, Seoul, Republic of Korea

[3] The CONVERSATIONALIST club, School of Stomatology, Shandong First Medical University, Jinan, Shandong, China

[4] Institute of Sports Medicine, Shandong First Medical University & Shandong Academy Medical Sciences, Tai'an, Shandong, China

[#] These authors contribute equally to this work.

[*] Authors to whom any correspondence should be addressed.

E-mail: Heng Bo Jiang, hengbojiang@foxmail.com; Lu Zhou, coolzhoulu@163.com

Fig S1. Original blots of ALP, BMPR2, ANG-1, MMP-9, β-actin of Figure 2D.

ALP

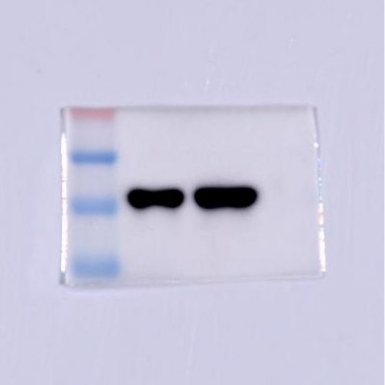

MMP-9

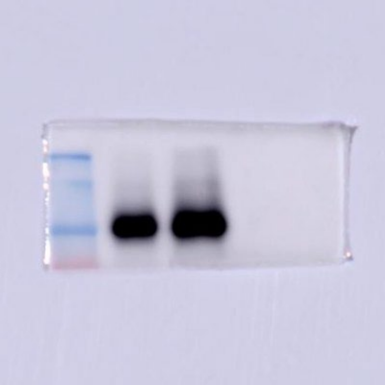

BMPR2

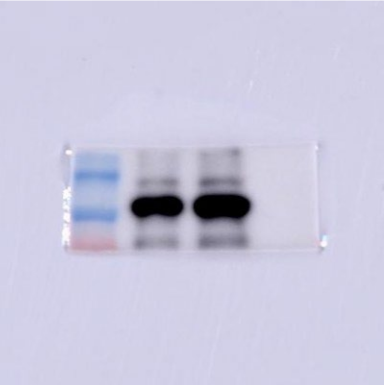

β-actin

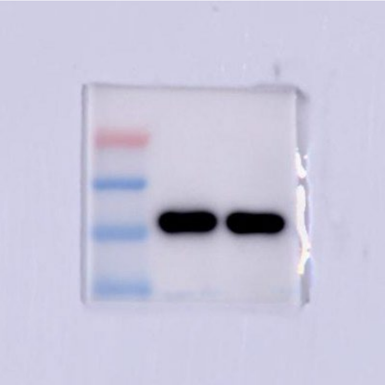

ANG-1

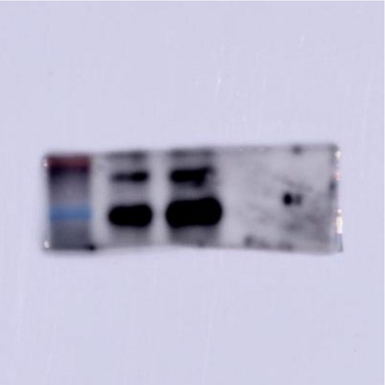

Fig S2. Original blots of YAP, P-YAP, HIF-1α, β-actin of Figure 4A.

YAP

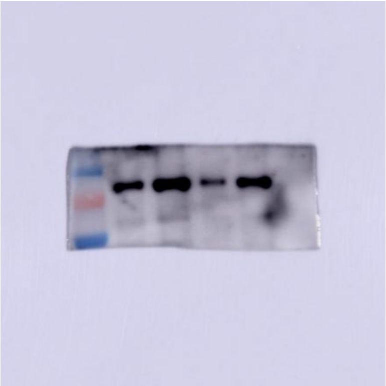

β-actin

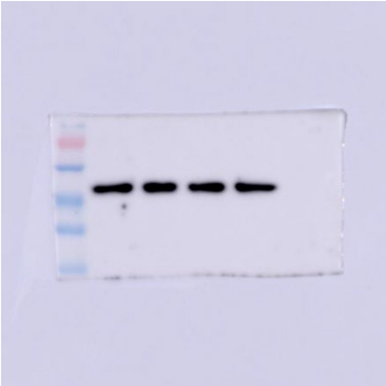

P-YAP

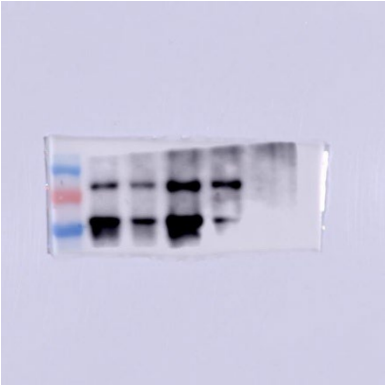

HIF-1α

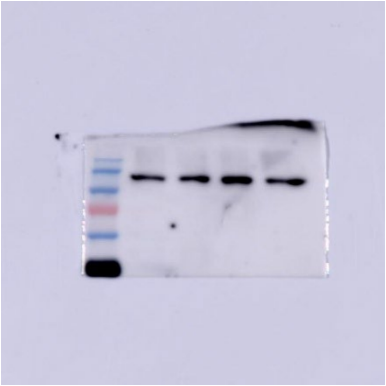

Fig S3. Original blots of HIF-1α, YAP, P-YAP, LATS1, β-actin of Figure 6D.

HIF-1α

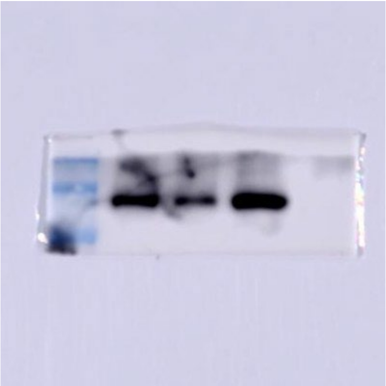

LATS1

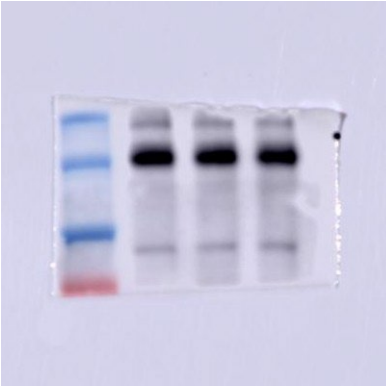

YAP

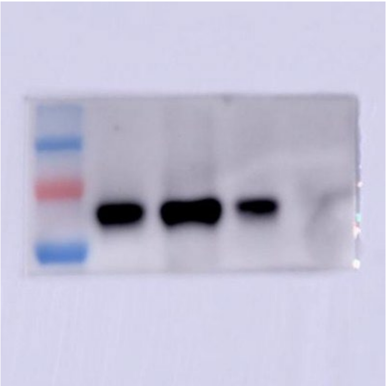

β-actin

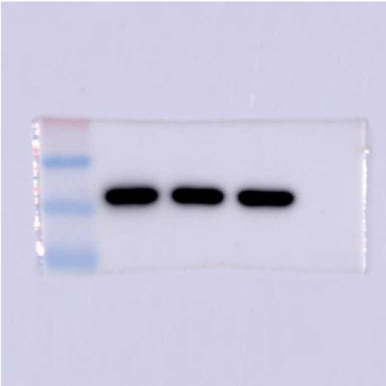

P-YAP

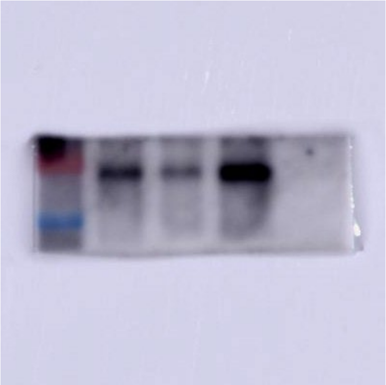

Supplement: Supplementary file 1 — Supplementary Material 1 [file 41598_2025_3015_MOESM1_ESM.pdf]
